# Social Media Use and Its Associations With Mental Health 9 Months After the COVID-19 Outbreak: A Cross-National Study

**DOI:** 10.3389/fpubh.2021.752004

**Published:** 2022-01-18

**Authors:** Hilde Thygesen, Tore Bonsaksen, Mariyana Schoultz, Mary Ruffolo, Janni Leung, Daicia Price, Amy Østertun Geirdal

**Affiliations:** ^1^Department of Occupational Therapy, Faculty of Health Sciences, Prosthetics and Orthotics, Oslo Metropolitan University, Oslo, Norway; ^2^VID Specialized University, Faculty of Health Studies, Department of Health, Oslo, Norway; ^3^Department of Health and Nursing Sciences, Faculty of Social and Health Sciences, Inland Norway University of Applied Sciences, Elverum, Norway; ^4^Faculty of Health and Life Sciences, Northumbria University, Newcastle upon Tyne, United Kingdom; ^5^School of Social Work, University of Michigan, Ann Arbor, MI, United States; ^6^Faculty of Health and Behavioural Science, The University of Queensland, Brisbane, QLD, Australia; ^7^Department of Social Work, Faculty of Social Sciences, Oslo Metropolitan University, Oslo, Norway

**Keywords:** coronavirus, cross-national study, pandemic, psychological distress, mental health, social distancing, social media, motives

## Abstract

**Background:**

The covid-19 pandemic has impacted the health and well-being of millions across the globe. Strict social distancing policies and periodic lockdowns has led to an increased reliance on alternative online means of communication, including social media.

**Objectives:**

to examine (i) social media use and mental health in the general population 9 months after the COVID-19 pandemic outbreak and (ii) mental health in relation to motives for and extent of social media use, while adjusting for sociodemographic variables.

**Methods:**

A cross-national online survey was conducted in Norway, UK, USA and Australia. Participants (*n* = 3,474) reported extent of and motives for social media use and completed the 12-item General Health Questionnaire. The data were analyzed by chi-square tests, one-way analyses of variance, and multiple linear regression analysis.

**Results:**

Poorer mental health was associated with using social media to decrease loneliness and for entertainment motives, while better mental health was associated with using social media for personal contact and maintaining relationships. Overall increased daily time on social media was associated with poorer mental health. The social media use variables were responsible for a substantial proportion of the outcome variance explained. These findings were consistent across the four countries, with only minor variations.

**Conclusions:**

Motives for using, and time spent using, social media were associated with the participants' mental health. Guidance and recommendations for social media usage to the general public for prevention and intervention for behavioral health may be beneficial.

## Introduction

The coronavirus pandemic has affected the lives of millions of people in various ways (1). Due to the high infection- and mortality rates caused by the virus, a number of strict measures have continued to be imposed by bodies of government across the world. A key element of these measures for individuals has been to reduce social contacts outside of the household or living situation. As a consequence, social distancing has become the new norm (2). The severity of the rules of social distancing has varied across regions and countries over time, as infection-rates have gone up or down. In general, however, people have been asked to reduce the number of contacts with individuals who are not a part of their household (3). Many schools, universities and workplaces have been closed or offered digital solutions only for students or employees (4). Also, many shops, restaurants and pubs have been closed for in-person gatherings, as well as many cultural- and social arenas, including cinemas and theaters, indoor sports activities and religious gatherings. Some countries have restricted travel or implemented additional screening requirements.

A number of studies have raised concerns about the coronavirus policies on people's lives and mental health, including its practical, social and financial aspects (5–7). For example, increased levels of anxiety, depression and loneliness has been reported (8). Other studies have shown a significant increase in emotional stress, also over time (6, 8–10). An important implication of the social distancing measures is the increased use of alternative means of communication, including social media (11). Social media is here understood as “applications that allow users to engage in virtual interactions, with broader or narrower audiences” (12).

Pre-coronavirus studies into the connections between social media use and mental health have revealed an ambiguous relationship (11, 13). Social media may be a source of entertainment, connection and information, while it may also fuel anxiety and stress (14). For example, daily use of social media has been associated with poorer mental health in young people (14, 15). The same two-sidedness is found in studies on social media use and mental health in the context of the current pandemic (16, 17). Although social media clearly has played an important role in connecting people during these times of extraordinary circumstances, the increased reliance on online means of communication and contact has also raised important concerns. For example, the overabundance of information on the coronavirus—some accurate, some not—prompted the warnings against the “infodemic” and anxiety caused by social media exposure (11, 18, 19). Also, as communication via social media does not fully compensate for face-to-face contact, prolonged periods of social distancing give rise to concerns about increased levels of loneliness (20).

Another important issue relates to association between motives for social media use and mental health. The literature on motives for social media use point at the many benefits that social media provide for its users (21–23). Interestingly, some of these studies point at the level of engagement with other (social media) users, as of particular importance in relation to mental health outcomes (24, 25). Active use of social media, where the person is in direct interaction with others, has been found to contribute to less loneliness and fewer mental health symptoms (22, 24). Passive use, on the other hand, such as scrolling through others' posts, has been associated with increased depressive symptoms, rumination and generally poorer mental health outcomes (24, 26).

Despite the growing literature on the coronavirus and its associations to mental health, studies on motives for social media use and its relationship to mental health are scarce. Specifically, studies need to expand from crude time-use measures of social media use, and need also to investigate whether associations with mental health are valid across countries and regions. Further, given the differences in social media use between sociodemographic groups, associations between social media use and mental health need to be corroborated by adjustments for sociodemographic background. All these requirements are addressed in the current cross-national study. The aim of this cross-national study was to examine (i) social media use and mental health in the general population 9 months after the COVID-19 pandemic outbreak, and (ii) examine mental health in relation to motives for and extent of social media use, while adjusting for sociodemographic variables.

## Methods

### Design and Procedures

The study is a cross-sectional survey conducted in Norway, USA, UK, and Australia. The online survey was distributed through social media platforms (i.e., Facebook, Twitter) in each of the involved countries between 24 October and 29 November 2020. A landing site for the survey was established at the researchers' universities; OsloMet - Oslo Metropolitan University, Norway; University of Michigan, USA; Northumbria University, UK; and the University of Queensland, Australia. The initiator of the project was AØG from OsloMet. Due to ethical considerations and permissions in each of the countries, each country had their own project lead. The survey was developed by the researchers in two languages; Norwegian and English, and was based on a previous survey conducted by the research group in the early phase (April 2020) of the pandemic outbreak (8, 27, 28). Language and cultural differences were considered during the survey development process.

### Inclusion and Exclusion

To be included in the study, participants had to be 18 years or older, understand Norwegian or English and live in Norway, USA, UK or Australia with access to the internet and electronic device. There were no additional exclusion criteria.

### Measures

#### Sociodemographic Characteristics

Sociodemographic variables included age group (18–29, 30–39, 40–49, 50–59, 60–69, 70 years and above), gender identity (male, female, other, prefer not to respond), highest completed education level (high school or associated/technical degree or lower, bachelor's degree, master's/doctoral degree), cohabitation (living with a spouse or partner, or not), and employment status (having full-time or part-time employment, or not).

#### Social Media Use

The participants were asked to indicate the amount of time they had spent on social media on a typical day during the last month. In line with the work of Ellison and co-workers, (29) response options were <10 min, 10–30 min, 31–60 min, 1–2 h, 2–3 h, and more than 3 h.

The participants were also asked about seven possible motives for using social media. These questions were adapted to a more general form based on Teppers et al. (30), whose study was concerned with one particular social media. The items were phrased: “Nowadays I use social media…” with the following endings: “to feel involved with what's going on with other people” (personal contact motive), “because it makes me feel less lonely” (decrease loneliness motive”), “so I don't get bored” (entertainment motive), “to keep in contact with my friends” (maintaining relationships motive), “because I dare say more” (social skills compensation motive), “to be a member of something” (social inclusion motive), and “to make new friends” (meeting people motive). Response options for these items were never (1), seldom (2), sometimes (3), often (4) and very often (5).

#### Mental Health

General Health Questionnaire 12 (GHQ-12) is widely used as a self-report measure of mental health (31, 32). A large number of studies in the general adult, clinical, work and student population have provided support for its validity across samples and contexts (32–36). Six items of the GHQ-12 are phrased positively (e.g., “able to enjoy day-to-day activities”), while six items are phrased as a negative experience (e.g., “felt constantly under strain”). For each item, the person indicates the degree to which the item content has been experienced during the two preceding weeks, using four response categories (“less than usual,” “as usual,” “more than usual” or “much more than usual”). Items are scored between 0 and 3, and positively formulated items are recoded prior to analysis. As a result, the GHQ-12 scale score range is 0–36, with higher scores indicating poorer mental health (more psychological distress). Cronbach's α for the GHQ-12 was 0.91.

### Statistical Analysis

Analyses were performed for the total sample and for each of the four countries. Descriptive analyses were performed for all included variables. Differences in GHQ scores between countries were investigated with independent *t*-tests and one-way analysis of variance (ANOVA). Multiple linear regression analysis was used to assess direct associations between each of social media use variables and mental health, while adjusting for all included variables. Variables were entered in two steps, representing sociodemographic variables: age group, gender, education level, cohabitation status and employment; and social media use: scores on personal contact motive, decrease loneliness motive, entertainment motive, maintaining relationships motive, social skills compensation motive, social inclusion motive, meeting people motive; and time spent on social media daily during the last month. Standardized beta weights (*β*) were reported as effect size, and according to Cohen (37), effect sizes about 0.10 were interpreted as small, effect sizes about 0.30 as moderate, and effect sizes about 0.50 as large. The outcome variance proportions explained by the models were reported. Statistical significance was set at *p* < 0.05. Missing values were handled by case-wise deletion.

### Ethics

The data collected in this study were anonymous. The researchers adhered to all relevant regulations in their respective countries concerning ethics and data protection. The study was approved by OsloMet (20/03676) and the regional committees for medical and health research ethics (REK; ref. 132066) in Norway, reviewed by the University of Michigan Institutional Review Board for Health Sciences and Behavioral Sciences (IRB HSBS) and designated as exempt (HUM00180296) in USA, by Northumbria University Health Research Ethics (HSR1920-080) in UK, and (HSR1920-080 2020000956) in Australia.

## Results

### Participants

Participants included 3,474 individuals from Norway (*n* = 547, 15.7%), USA (*n* = 2130, 61.3%), UK (*n* = 640, 18.4%) and Australia (*n* = 157, 4.5%). In the total sample, there was a spread across age groups, with a lower proportion of the oldest participants (above 70 years). There were less men than women (22.2% men vs. 73.3% women). Seventy-one percent had a bachelor's degree or higher levels of education. Full-time or part-time employment was held among 66.3%, while 58.7% lived with a spouse or partner.

### Mental Health in Sample Subgroups

Table 1 displays the levels of mental health according to sample subgroups in the total sample and for each of the four countries. In the total sample, mental health was better in the older age groups, and men reported better mental health than women. Participants with higher levels of education reported better mental health compared to those with lower education levels, while those living with spouse or partner reported better mental health than their counterparts. Mental health was not significantly different between participants with and without employment.

**Table 1:** GHQ scores by participant characteristics in the total sample and in each country.

	**Total sample**	**USA**	**UK**	**Norway**	**Australia**
**Characteristics**	**M (SD)**	**M (SD)**	**M (SD)**	**M (SD)**	**M (SD)**
**Age group**					
18–29 years	18.9 (6.8)^***^	18.5 (6.8)^***^	20.7 (6.9)^***^	18.4 (6.2)^***^	17.7 (7.3)
30–39 years	17.4 (6.5)	17.5 (6.4)	17.9 (6.5)	16.8 (7.0)	15.1 (5.6)
40–49 years	16.5 (6.7)	16.6 (6.2)	17.8 (7.2)	15.1 (7.2)	14.2 (6.5)
50–59 years	15.4 (6.7)	14.9 (6.4)	17.9 (6.7)	13.7 (6.6)	15.0 (7.1)
60–69 years	14.5 (6.3)	14.4 (5.8)	16.8 (7.1)	13.1 (7.0)	15.1 (7.0)
70 years +	12.9 (5.8)	13.2 (5.5)	15.0 (7.0)	10.9 (5.6)	13.2 (6.8)
**Gender identity**					
Male	14.8 (7.1)^***^	14.7 (6.9)^***^	17.5 (7.6)	13.0 (7.2)^**^	14.3 (6.0)
Female	16.9 (6.6)	16.9 (6.2)	18.6 (6.8)	15.4 (6.9)	15.1 (7.0)
**Education level**					
High school/tech. degree or lower	16.8 (7.4)^***^	16.0 (7.0)^**^	18.8 (7.6)	17.0 (7.7)^***^	16.5 (7.9)
Bachelor's degree	16.8 (6.9)	17.1 (6.8)	18.7 (6.7)	14.6 (7.1)	14.9 (6.7)
Master's/doctoral degree	15.7 (6.2)	16.0 (6.6)	17.4 (6.6)	13.8 (6.2)	14.6 (6.3)
**Cohabitation**					
Yes	15.8 (6.5)^***^	16.1 (6.3)^**^	17.2 (6.6)^***^	13.9 (6.6)^***^	13.5 (5.6)^**^
No	17.3 (7.2)	16.8 (7.0)	20.0 (7.2)	16.4 (7.3)	17.5 (7.8)
**Employment**					
Full-time or part-time	16.5 (6.6)	16.7 (6.3)^**^	18.0 (6.8)	14.4 (6.5)^**^	15.3 (6.7)
No employment	16.3 (7.3)	15.6 (6.9)	19.2 (7.4)	16.1 (8.1)	15.3 (7.1)

*Statistical tests are one-way ANOVA F-test (age groups and education level) and independent t-tests (all other variables). p-values refer to differences within the total sample and within each of the subsamples. Cohabitation refers to “living with spouse or partner.” Higher GHQ scores indicate poorer mental health*.

** *p < 0.01*,

*** *p < 0.001*.

The overall pattern of better mental health in the older age groups was consistent across all countries, with significant differences between older and younger age groups found for USA, UK and Norway. Among participants in the USA, mental health was significantly better among those not employed, compared to their employed counterparts, whilst in Norway, better mental health was found among those who were employed.

### Social Media Use and Mental Health

The mean scores for each of the seven purposes or motives for social media use are reported in Table 2. In the total sample, the highest mean score was shown for the motive for maintaining relationships, while the motive for meeting people was least endorsed. Sixty-two percent of the sample reported using social media for at least 1 h daily, while 21% reported using social media for more than 3 h daily.

**Table 2:** Social media use motives and time spent in the total sample and in the four countries.

**Motives**	**Total sample**	**USA**	**UK**	**Norway**	**Australia**	***p***
	**M (SD)**	**M (SD)**	**M (SD)**	**M (SD)**	**M (SD)**	
Personal contact	3.5 (1.0)	3.6 (1.1)	3.4 (1.1)	3.3 (1.0)	3.4 (1.0)	<0.001
Decrease loneliness	2.7 (1.3)	2.7 (1.3)	2.6 (1.2)	2.5 (1.2)	2.4 (1.2)	<0.001
Entertainment	3.5 (1.1)	3.6 (1.1)	3.5 (1.2)	3.5 (1.2)	3.1 (1.2)	<0.001
Maintaining relationships	3.7 1.0)	3.7 (1.0)	3.8 (1.0)	3.6 (1.0)	3.6 (1.0)	<0.05
Social skills compensation	2.0 (1.1)	2.1 (1.2)	1.8 (1.0)	1.5 (0.8)	1.8 (1.0)	<0.001
Social inclusion	2.3 (1.2)	2.3 (1.2)	2.2 (1.2)	2.5 (1.2)	2.4 (1.2)	<0.01
Meeting people	1.6 (0.8)	1.6 (0.9)	1.6 (0.9)	1.5 (0.8)	1.5 (0.8)	<0.01
**Daily time on social media**	***n*** **(%)**	***n*** **(%)**	***n*** **(%)**	***n*** **(%)**	***n*** **(%)**	**<0.001**
<10 min	77 (2.2)	24 (1.3)	14 (2.7)	36 (6.6)	3 (2.3)	
10–30 min	272 (7.8)	145 (8.1)	47 (9.1)	69 (12.6)	11 (8.5)	
$\frac{1}{2}$-1 h	492 (14.2)	267 (14.9)	90 (17.5)	108 (19.7)	27 (20.9)	
1–2 h	859 (24.7)	500 (27.9)	131 (25.4)	189 (34.6)	39 (30.2)	
2–3 h	567 (16.3)	429 (23.9)	107 (20.8)	2 (0.4)	29 (22.5)	
3 h or more	718 (20.7)	1,794 (23.9)	515 (24.5)	547 (26.1)	129 (15.5)	
**Mental health**	**M (SD)**	**M (SD)**	**M (SD)**	**M (SD)**	**M (SD)**	***p***
GHQ score	16.4 (6.8)	16.4 (6.6)	18.3 (7.0)	14.9 (7.0)	15.2 (6.9)	<0.001

*Response options for the motive items were never (1), seldom (2), sometimes (3), often (4) and very often (5). In total, 2,980 (85.9%) of the participants responded to the question about daily time spent on social media. p-values, indicating the probability of between-country differences in the population, refer to the ANOVA F-test (motives and mental health) and the Chi-square test (daily time on social media)*.

There were statistically significant differences between the four countries regarding the participants' endorsement of motives for social media use. Across countries, though, there were high endorsements for the personal contact, entertainment and maintaining relationships motives (at similar levels), while the lowest level of endorsement was found for the meeting people motive. Mental health was also significantly different between the four countries. Participants in the UK had poorer mental health compared to all other countries, while participants in the USA also had poorer mental health compared to participants in Norway. The levels were not significantly different between Norway and Australia.

### Associations Between Mental Health and Social Media Use

Adjusted associations between social media use and mental health are displayed in Table 3. In the multiple regression analysis for the total sample, better mental health was associated with higher endorsement of the personal contact motive (*β* = −0.07, *p* < 0.001) and the maintaining relationships motive (*β* = −0.10, *p* < 0.001). Poorer mental health was associated with higher endorsement of the decrease loneliness motive (*β* = 0.29, *p* < 0.001) and the entertainment motive (*β* = 0.13, *p* < 0.001). In addition, more time spent on social media daily was associated with poorer mental health (*β* = 0.07, *p* < 0.001). The variables concerned with social media use accounted for 12.0% of the GHQ variance. Among the sociodemographic (control) variables, better mental health was associated with higher age, male gender, having higher education and having employment.

**Table 3:** Adjusted associations with GHQ scores in the total sample and in the four countries.

**Independent variables**	**Total sample**	**USA**	**UK**	**Norway**	**Australia**
**Sociodemographic variables**	**β**	**β**	**β**	**β**	**β**
Higher age	−0.17^***^	−0.17^***^	−0.05	−0.22^***^	−0.08
Female gender	0.06^***^	0.06*	0.07	0.05	−0.03
Higher education level	−0.04	−0.03	−0.03	−0.02	0.00
Living with spouse/partner	−0.04^*^	0.00	−0.14^**^	−0.03	−0.22^*^
Having employment	−0.05^**^	−0.02	−0.07	−0.16^***^	−0.04
***R*** **^2^ change**	9.9%^***^	9.2%^***^	7.6%^***^	18.4%^***^	10.6%*
**Social media use**					
Personal contact motive	−0.07^**^	−0.07^*^	−0.08	−0.07	−0.05
Decrease loneliness motive	0.29^***^	0.34^***^	0.11^*^	0.27^***^	0.18
Entertainment motive	0.13^***^	0.11^***^	0.21^***^	0.17^**^	0.20
Maintaining relationships motive	−0.10^***^	−0.09^**^	−0.16^**^	−0.14^**^	−0.04
Social skills compensation motive	0.03	−0.01	0.08	0.15^**^	0.03
Social inclusion motive	0.03	0.05	−0.08	0.09	0.15
Meeting people motive	−0.03	−0.05	−0.00	−0.07	−0.11
Time spent on social media daily	0.07^***^	0.09^***^	0.07	0.01	0.15
***R*** **^2^ change**	12.0%^***^	14.3%^***^	7.8%^***^	14.9%^***^	17.6%**
**Explained variance**	21.8%^***^	23.5%^***^	15.3%^***^	33.3%^***^	28.2%^***^

*Standardized beta values (β) indicate strength of associations adjusted for all included variables*.

*** *p < 0.001*,

** *p < 0.01*,

* *p < 0.05*.

Between the four countries, the associations between social media use and mental health were relatively uniform, but with varying effect sizes and probability measures. The association between higher endorsement of the personal contact motive and better mental health was only significant among the participants from USA. The decrease loneliness motive was more strongly associated with poorer mental health among participants from USA and Norway, compared to participants from UK and Australia. The entertainment motive was more strongly associated with poorer mental health among participants from UK and Norway, while less strongly associated among participants from the USA. The maintaining relationships motive was weakly, but significantly associated with better mental health among participants from USA, UK and Norway. Higher endorsement of the social skills compensation motive was associated with poorer mental health only among participants from Norway. The social inclusion and meeting people motives were not significantly associated with mental health among participants in any of the countries. More time spent on social media during a typical day was significantly associated with poorer mental health only among the participants from USA. The social media variables accounted for varying proportions of GHQ variance between the countries: between 7.8% in the UK and 17.6% in Australia.

## Discussion

The aims of this study were to examine the associations between social media use and mental health in the general population 9 months after the COVID-19 pandemic outbreak, and to examine mental health in relation to motives for- and time spent on social media use, while adjusting for sociodemographic variables. In the adjusted model for the whole sample, poorer mental health was associated with using social media to decrease loneliness and for entertainment motives, while better mental health was associated with using social media for personal contact and maintaining relationships. Overall increased daily time on social media was associated with poorer mental health. These findings were relatively consistent across the countries that participated in the survey, with only minor variations. In sum, we found that motives for and time spent on social media use were responsible for a substantial proportion of the variance explained in the sample's mental health 9 months into the COVID-19 pandemic.

Clearly, social media is an important part of many people's lives. Currently, it is estimated that more than 1.8 billion people use Facebook on a daily basis (38), while the corresponding numbers for Instagram and Twitter are 1.1 billion (39) and 192 million (40), respectively. Although the popularity of different social media platforms varies over time and across countries, social media use in general is on the increase (11, 41). This gives rise to questions of the kinds of values that social media bring about for its users.

Our finding, that high daily use of social media was associated with poorer mental health, corresponds with other research in the field (42, 43). Recent studies show that this pattern is also found in the current context of the pandemic (9, 13, 16, 17, 20, 44–47). These findings may lead to the assumption that social media use—in itself—may be detrimental to mental health. However, a reversed causality is equally possible. Poor mental health may lead to more time spent on social media.

Social media use is complex, including the relations between motives for use and mental health. Our study showed that poorer mental health was associated with using social media to decrease loneliness and for entertainment purposes. In contrast, better mental health was associated with using social media for personal contact and maintaining relationships motives. These differences in motives can be seen to coincide with the distinctions between passive and active social media use (26, 48–50). Examples of passive use of social media are scrolling through news feeds or browsing photographs of friends. Passive use of social media has been associated with negative mental health outcomes, including depression, fatigue and a reduction in psychological well-being (26, 49). Active social media users, on the other hand, share life experiences, create text, and respond frequently to other users (50). According to Lin and co-workers (48), active users often experience higher social support, which helps them to have a more favorable attitude toward themselves. The results of our study, that better mental health was associated with using social media for personal contact and maintaining relationships correspond to these findings. On the contrary, the use of social media for the purposes of decreasing loneliness and for entertainment, fits with a passive user profile and is therefore logically related to poorer mental health outcomes.

A concern raised is that passive use of social media seems to dominate (24, 51). This suggests that many people spend much of their time on social media engaging in behavior that may undermine their well-being. A timely question is why this may be the case. There is a growing literature that suggests that social media have addictive properties (41, 49). An element of addiction may explain why some people behave in ways they realize can be harmful to themselves. Also, it may be possible that some social media users are not aware of the negative implications. According to Lisitsa et al. (24), the current pandemic and the combination of more social media use and well as higher stress levels, are likely to encourage avoidance behaviors, such as passive scrolling rather than active engagement with others online. In particular, this may be the case for young adults, who engage more with social media than people in older age groups. Also, passive users of social media may be more susceptible to the negative mental health effects related to the spread of misinformation and fake news that are currently circulated amongst their social media networks (11, 16, 46).

The complex relationships between social media use, its motives and mental health imply no easy solutions. On the one hand, this study provides support for the notion that extensive use of social media is related to poorer mental health. On the other hand, the relation between social media use and mental health appears to be contingent on how and why social media is used. Therefore, to support mental health, critical questions for self-reflection among social media users may go beyond the “how much” question to include inquiry into the “for what purpose(s).” Social media are not inherently bad, but as they contribute to shape people's lives, a critical, self-reflective stance toward their use is required.

## Study Limitations

Respondents were invited to participate through electronic social media. With social media being an aspect for individuals to potentially engage with others, the responses are not inclusive of individuals that do not utilize social media. As also seen from the skewed gender distribution, the sample included in the study is therefore not representative of the general population. This limits the ability to generalize the results to the general population.

A limitation of the study is that we did not take into consideration that already established mental health problems could exacerbate problems related to social distancing measures during the pandemic, with possible consequences for the use of social media. Also, a limitation of our study is that the estimation of time spent using social media is based on self-report only, which does not necessarily reflect actual time spent on social media. It is important to note that the associations between social media use and mental health may be moderated by variables such as social- or community support, cohabitation status and employment. In addition, it is possible that those with higher levels of social capital and support may rely less on social media than people in other segments of the population. Thus, future studies may investigate these associations within and between specified population subgroups. Future studies may also use more targeted self-report measures, related for example to depression and anxiety, to obtain information about mental health.

Due to the cross-sectional design of the study, we do not know whether those who often used social media to reduce loneliness had already improved in their mental health, or if social media use had exacerbated their psychological distress. Future studies that use a longitudinal design can provide data on changes in psychosocial health within the same people following social media use. In addition, future studies that evaluate mental health interventions in light of COVID-19-related restrictions are needed to address the increased depression and anxiety observed across populations due to the pandemic. A final point is that future studies including other countries and populations would be valuable, as associations between social media use and mental health may vary between different contexts.

## Conclusion

The individual's motives for using social media and the time spend on social media is associated with one's mental health. Using social media as a coping strategy during restrictions to maintain human relationships appears to be related to better mental health. However, when individuals use social media for entertainment or to reduce loneliness, higher levels of stress and anxiety emerge. The more time spent on social media regardless of the motive for using social media was associated with poorer mental health outcomes. Guidance and recommendations for social media usage to the general public for prevention and intervention for behavioral health may be beneficial.

## Data Availability Statement

The raw data supporting the conclusions of this article will be made available by the authors, without undue reservation.

## Ethics Statement

The studies involving human participants were reviewed and approved by OsloMet (20/03676) and the Regional Committees for Medical and Health Research Ethics (REK; ref. 132066) in Norway, reviewed by the University of Michigan Institutional Review Board for Health Sciences and Behavioral Sciences (IRB HSBS) and designated as exempt (HUM00180296) in USA, by Northumbria University Health Research Ethics (HSR1920-080) in UK, and (HSR1920-080 2020000956) in Australia. The patients/participants provided their written informed consent to participate in this study.

## Author Contributions

HT, TB, MS, MR, DP, JL, and AG: conceptualization, methodology, validation, investigation, and writing—review and editing. HT and TB: formal analysis and writing—original draft preparation. TB and AG: data curation. AG: project administration. All authors contributed to the article and approved the submitted version.

## Conflict of Interest

The authors declare that the research was conducted in the absence of any commercial or financial relationships that could be construed as a potential conflict of interest.

## Publisher's Note

All claims expressed in this article are solely those of the authors and do not necessarily represent those of their affiliated organizations, or those of the publisher, the editors and the reviewers. Any product that may be evaluated in this article, or claim that may be made by its manufacturer, is not guaranteed or endorsed by the publisher.
